# Assessment of Acute Side Effects Among 3D-Concurrent Radiotherapy With Cisplatin-Treated Head and Neck Cancer Patients

**DOI:** 10.7759/cureus.44238

**Published:** 2023-08-28

**Authors:** Hina Mehmood, Talha Laique, Ayesha Ahmad, Rizwan Ahmad

**Affiliations:** 1 Dermatology, James Paget University Hospital, Great Yarmouth, GBR; 2 Pharmacology, Lahore Medical and Dental College, Lahore, PAK; 3 Radiation Oncology, Ghurki Trust Teaching Hospital Lahore, Lahore, PAK; 4 Ophthalmology, James Paget University Hospital, Great Yarmouth, GBR

**Keywords:** acute side effects, cisplatin and 3d-concurrent radiotherapy, dermatitis, xerostomia, head and neck cancer

## Abstract

Introduction: Three-dimensional conformal radiation therapy has become one of the basic components of multidisciplinary treatment for head and neck cancer. Generally, patients with squamous-cell carcinoma of the head and neck receive cisplatin-based chemoradiation.

Aims: In the current project, the goal was to assess 3D-CRT with cisplatin-induced acute side effects (dermatitis plus xerostomia) among head and neck cancer patients.

Methodology: This descriptive case series was held at the Institute of Nuclear Medicine and Oncology, Lahore, Pakistan, with an enrollment of 106 head and neck cancer patients following the hospital’s ethical approval. All patients received 3D-CRT with concurrent cisplatin chemotherapy according to the oncology treatment protocol at the Institute of Nuclear Medicine and Oncology. The evaluation of enrolled patients was done during treatment at a weekly interval and at one-month post-radiation. Stage 3 patients (17.9%) received chemo-radiation therapy with 40 mg/m^2^ cisplatin once weekly for seven weeks. All patients received 70 grays in 35 fractions with two grays per fraction over the course of seven weeks following a standard protocol. All enrolled cases had biopsy-proven squamous cell carcinoma of the head and neck. IBM Corp. Released 2015. IBM SPSS Statistics for Windows, Version 23.0. Armonk, NY: IBM Corp. analyzed the data. Chi-square and Fisher's exact tests were applied, while a p-value ≤ 0.05 was taken as statistically significant.

Results: All patients developed acute skin changes (dermatitis plus xerostomia) as a side effect of radiation therapy, with cisplatin having different grades during treatment until seven weeks. However, these changes improved and became less severe in terms of grade after one month of post-treatment among all patients.

Conclusion: It was concluded that 3D-CRT was associated with dermatitis and xerostomia during and immediately after follow-up, even though the treatment response was good. However, clinical signs and symptoms improved, indicating that radiation therapy is a relatively safe treatment modality among cancer patients. Moreover, 40 mg/m^2^ cisplatin once weekly for seven weeks resulted in better loco-regional control and survival among advanced-stage head and neck cancer patients as a part of treatment. Although, higher doses of cisplatin (100 mg/m^2^ ) every three weeks have more harmful acute side effects and delay treatment for patients due to poor compliance.

## Introduction

Head and neck cancer impacted lives badly, both physically and mentally. Unfortunately, the oral cavity, nasopharynx, or hypopharynx are common targets for disease, as revealed by a literature review. It may involve the tongue, posterior and lateral pharynx, mucosa of the oral cavity, and pharynx [1]. Among all cancer types, squamous cell carcinomas constitute the majority (90%) of the cases globally. Today’s one of the health problems is head and neck cancer (HNC) globally. Its management is a challenge for the health community. Head and neck cancer (HNC) radiation oncologists (ROs) help in curing patients through a multi-disciplinary approach to reduce toxic effects [2]. Head and neck cancers have a high mortality rate and cause almost 300,000 deaths each year, as estimated by one previous survey globally. These cancers are aggressive malignancies with an annual incidence of more than 550,000 cases in Asia, according to one study [3]. Unfortunately, this disease is gender biased, affecting males more in comparison to females, with ratios ranging from 2:1 to 4:1 [4,5].

Patients with early-stage disease usually have vague signs and symptoms. They depend on the primary site involved in disease [6]. Patients have non-healing ulcers with pain if the oral cavity is involved, whereas oropharynx involvement is present as a sore throat, chronic dysphagia, and otalgia. By contrast, patients usually have a neck mass with supraglottic tumors due to the advancement of the disease [6].

In developing countries and Asia, the predisposing factors include alcohol, tobacco consumption, and betel nut chewing, which contribute towards its development [7]. Alcohol and tobacco consumption have a synergistic effect as carcinogens when combined. Furthermore, women have adopted the male pattern of alcohol and tobacco consumption in recent years. Other risk factors that contributed to the development of oral cavity cancers include genetic susceptibility, viral infections, radiotherapy, and poor oral hygiene. Treatment failure occurs because of poor compliance with treatment, treatment side effects, and traditional medicines involving herbal and hakeem systems [8,9].

The purpose of concurrent radiotherapy is to kill tumor cells maximally. Although 3D-CRT is linked with several side effects like dermatitis and xerostomia among patients. In light of the increasing burden of head and neck cancer and the difficulty in treating this disease due to the various side effects of 3D-CRT, we planned the current study to assess 3D-CRT with cisplatin-induced changes in skin physiology (Dermatitis and Xerostomia) among cancer patients. This study helped us identify the acute side effects of 3D-CRT in terms of dermatitis and xerostomia among the local Pakistani population. This study added information to the existing literature review so we could make a proper strategy regarding the side effects of radiation therapy. Once side effects are managed and addressed properly for cancer patients, adequate treatment will decrease the burden of this disease. A proper treatment strategy will improve treatment compliance among patients.

## Materials and methods

This descriptive case series was held at the Institute of Nuclear Medicine and Oncology, Lahore, Pakistan, following the hospital’s ethical approval. Patients (n = 106) received concurrent 3D-radiotherapy with cisplatin according to the current treatment protocol [10]. Patients who were reluctant and had a second malignancy or pregnancy were excluded. Written informed consent was obtained from each patient. All patients were given radiotherapy or chemo-radiotherapy as per clinician advice and hospital protocol. All patients were evaluated at pre-radiation time, at weekly intervals during treatment, and at 11 weeks from the first radiation fraction. In the present study, 82.1% of patients (stages 1 and 2) received only radiation therapy, while stage 3 patients (17.9%) received chemo-radiation therapy with 40 mg/m2 cisplatin once weekly for seven weeks [11]. All patients received 70 grays in 35 fractions with two grays per fraction over the course of seven weeks following a standard protocol. All enrolled cases had biopsy-proven squamous cell carcinoma of the head and neck. 

A non-probability, consecutive sampling technique was used. Both male and female patients, ages 25 to 75, were enrolled in stage I-III of head and neck cancer with ECOG status 1 and 2. All baseline parameters were enrolled in a performance at the time of enrollment. Strict monitoring was done for patients with acute side effects.

After initial work-up, staging was done to evaluate the extent of disease by using the American Joint Commission on Cancer's (AJCC) TMN staging system. Universally, staging systems for cancers include the TNM classification system based on anatomical information. At the time of diagnosis or after surgery, tumor size or location (T), regional lymph node involvement (N), and distant metastases (M) are the indicators for disease metastasis as well as its prognosis. Important points regarding TNM staging are: A) The T staging for head and neck cancers differs according to the primary site. B) The N staging is common for all subsites except the nasopharynx. C) The M staging is common to all sites (Table 1).

**Table 1:** TNM staging for all subsites except nasopharynx LN= Lymph nodes

Classification	Characteristics
T_1_	Tumor ≤ 12 cm in greatest dimensions
T_2_	Tumor > 2cm but < 4cm in greatest dimensions
T_3_	Tumor > 4cm in greatest dimensions
T_4_	Tumor invades adjacent structures
N_0_	No regional LN
N_1_	Single ipsilateral LN ≤ 3cm
N_2a_	Single ipsilateral LN,3-6 cm
N_2b_	Multiple ipsilateral LN, none >6cm
N_2c_	Bilateral or contralateral LN, none >6cm
N_3_	Any LN, none >6cm
M_0_	No distant metastasis
M_1_	Distant metastasis

After RT or accidental exposures, acute changes after RT treatment in cancerous patients appear within 90 days. Generalized erythema usually appears within hours after RT but vanishes within days. The second phase of severe erythema appears within two weeks after dosing without any epidermal changes due to cytokines (Table 2). 

**Table 2:** Criteria for grading dermatitis by WHO

Adverse Events	Grades			
	1	2	3	4
Dermatitis	Faint erythema or dry desquamation	Moderate erythema, edema, desquamation confined to skin folds	Desquamation ≥ 1.5cm not confined to skin folds, pitting edema	Skin necrosis or ulceration.

There are mainly three main salivary glands (parotid, sublingual, and submandibular) surrounding the mouth, which secrete their secretions into the mouth via ducts. These three primary glands secrete around 90% of total salivary secretions. As a result of 3D-CRT, the patient's saliva becomes thick and tenacious. However, if RT continues, mucous cells, as well as the quantity of saliva, will decrease (Table 3).

**Table 3:** Criteria for grading Xerostomia by WHO

Adverse Effect	Grade 0	Grade 1	Grade 2	Grade 3	Grade 4	Grade 5
Xerostomia	None	Slight dryness of mouth, good response on stimulation	Moderate dryness of mouth, poor response on stimulation	Complete dryness of mouth, no response on stimulation	Fibrosis	Death

IBM Corp. Released 2015. IBM SPSS Statistics for Windows, Version 23.0. Armonk, NY: IBM Corp. analyzed the data. Chi-square and Fisher's exact tests were applied, while a p-value ≤ 0.05 was taken as statistically significant. Mean± SD presented quantitative parameters. Frequencies and percentages represented qualitative parameters like gender, cancer stage, and ECOG status (performance scale) of patients.

## Results

Baseline parameters (age and dose of radiation) were noted at the time of enrollment in terms of mean± SD (Table 4). 

**Table 4:** Baseline quantitative parameters

	Mean ± SD	Minimum	Maximum
Age (years)	57.8 ± 8.3	39	70
Total radiation dose (Grays)	62.8 ± 7.4	30	70

The distribution of qualitative parameters among head and neck cancer patients (Table 5).

**Table 5:** Distribution of participants with respect to qualitative parameters

Variable	Category	Frequency	Percentage (%)
Gender	Male	44	41.5
	Female	62	58.5
Cancer	Stage 1	41	38.7
	Stage 2	46	43.4
	Stage 3	19	17.9
ECOG	Poor	67	63.2
	Good	39	36.8
Oral hygiene	Poor	26	24.5
	Average	28	26.4
	Good	52	49.1

An improvement in grades of dermatitis appeared among all patients post-treatment (Table 6). Radiation Therapy Oncology Group (RTOG) and the European Organization for Research and Treatment of Cancer (EORTC) Toxicity criteria were also used in accordance with WHO criteria.

**Table 6:** Distribution of patients during and after treatment into different grades of dermatitis

Week	Grade 0	Grade 1	Grade 2	Grade 3	Grade 4	Grade 5
1^st^	98 (92.5%)	8 (7.5%)	0 (0%)	0 (0%)	0 (0%)	0 (0%)
2^nd^	67 (63.2%)	39 (36.8%)	0 (0%)	0 (0%)	0 (0%)	0 (0%)
3^rd^	8 (7.5%)	90 (84.5%)	8 (7.5%)	0 (0%)	0 (0%)	0 (0%)
4^th^	0 (0%)	67 (63.2%)	31 (29.2%)	8 (7.5%)	0 (0%)	0 (0%)
5^th^	0 (0%)	14 (13.2%)	84 (79.2%)	8 (7.5%)	0 (0%)	0 (0%)
6^th^	0 (0%)	0 (0%)	51 (48.1%)	47 (44.3%)	8 (7.5%)	0 (0%)
7^th^	0 (0%)	6 (5.7%)	8 (7.5%)	84 (79.2%)	8 (7.5%)	0 (0%)
11^th^	0 (0%)	14 (13.2%)	53 (50%)	39 (36.8%)	0 (0%)	0 (0%)

There is an insignificant difference among different dermatitis grades based on gender at different weeks of treatment (Table 7). Radiation Therapy Oncology Group (RTOG) and the European Organization for Research and Treatment of Cancer (EORTC) Toxicity criteria were also used in accordance with WHO criteria.

**Table 7:** Different dermatitis grades on the basis of gender

Week	Gender	Grade 0	Grade 1	Grade 2	Grade 3	Grade 4	Grade 5	p-value
1^st^	Male	40(90.9%)	4(9.1%)	Nil	Nil	Nil	Nil	0.716
	Female	58(93.5%)	4(6.5%)	Nil	Nil	Nil	Nil	
4^th^	Male	0 (0%)	26(59.1%)	14(31.8%)	4(9.1%)	0 (0%)	0 (0%)	0.775
	Female	0 (0%)	41(66.1%)	17(27.4%)	4(6.5%)	0 (0%)	0 (0%)	
7^th^	Male	0 (0%)	1(2.3%)	4(9.1%)	35(79.5%)	4(9.1%)	0 (0%)	0.625
	Female	0 (0%)	5(8.1%)	4(6.5%)	49(79.0%)	4(6.5%)	0 (0%)	
11^th^	Male	0 (0%)	5(11.4%)	21(47.7%)	18(40.9%)	0 (0%)	0 (0%)	0.734
	Female	0 (0%)	9(14.5%)	32(51.6%)	21(33.9%)	0 (0%)	0 (0%)	

At the seventh week of treatment, xerostomia of grade 4 was observed in 57 (53.8%) patients and improved afterwards (Table 8). Radiation Therapy Oncology Group (RTOG) and the European Organization for Research and Treatment of Cancer (EORTC) Toxicity criteria were also used in accordance with WHO criteria.

**Table 8:** Distribution of patients according to different grades of xerostomia

Week	Grade 0	Grade 1	Grade 2	Grade 3	Grade 4	Grade 5
1^st^	78 (73.6%)	28 (26.4%)	0 (0%)	0 (0%)	0 (0%)	0 (0%)
2^nd^	78 (73.6%)	21 (19.8%)	7 (6.6%)	0 (0%)	0 (0%)	0 (0%)
3^rd^	49 (46.2%)	29 (27.4%)	28 (26.4%)	0 (0%)	0 (0%)	0 (0%)
4^th^	10 (9.4%)	68 (64.2%)	18 (17%)	10 (9.4%)	0 (0%)	0 (0%)
5^th^	0 (0%)	29 (27.4%)	49 (46.2%)	18 (17%)	10 (9.4%)	0 (0%)
6^th^	0 (0%)	10 (9.4%)	39 (36.8%)	30 (28.3%)	27 (25.5%)	0 (0%)
7^th^	0 (0%)	0 (0%)	29 (27.4%)	20 (18.9%)	57 (53.8%)	0 (0%)
11^th^	0 (0%)	0 (0%)	69 (65.1%)	37 (34.9%)	0 (0%)	0 (0%)

There was an insignificant difference among different xerostomia grades on the basis of gender at different weeks of treatment (Table 9).

**Table 9:** Different xerostomia grades on the basis of gender

Week	Gender	Grade 0	Grade 1	Grade 2	Grade 3	Grade 4	Grade 5	p-value
1^st^	Male	33 (75.0%)	11 (25.0)	0 (0%)	0 (0%)	0 (0%)	0 (0%)	0.781
	Female	45 (72.6)	17 (27.4)	0 (0%)	0 (0%)	0 (0%)	0 (0%)	
4^th^	Male	4 (9.1%)	65.9%)	8 (18.2%)	3 (6.8%)	0 (0%)	0 (0%)	0.924
	Female	6 (9.7%)	39 (62.9%)	10 (16.1%)	7 (11.3%)	0 (0%)	0 (0%)	
7^th^	Male	0 (0%)	0 (0%)	13 (29.5%)	7 (15.9%)	24 (54.5%)	0 (0%)	0.783
	Female	0 (0%)	0 (0%)	16 (25.8%)	13 (21.0%)	33 (53.2%)	0 (0%)	
11^th^	Male	0 (0%)	0 (0%)	28 (63.6%)	16 (36.4%)	0 (0%)	0 (0%)	0.791
	Female	0 (0%)	0 (0%)	41 (66.1%)	21 (33.9%)	0 (0%)	0 (0%)	

## Discussion

Unfortunately, the high prevalence of head and neck cancer among our population has affected our pupils badly, both mentally and physically. However, Pakistan being a developing country with limited human resources, this health issue remained untouched in our setups. Thus, we examined the safety of concurrent 3D-RT in non-metastatic cancer patients enrolled in our clinical setup at the Institute of Nuclear Medicine and Oncology [12]. Causes of therapeutic failure for both chemotherapy and radiation therapy are false beliefs about drugs being prescribed in oncology centers and parallel treatment systems, e.g., traditional medicines, that drift them away from taking treatment for the desired duration [8,9]. Thus, this attitude increases the burden of disease in society. Moreover, they have to face severe acute as well as chronic toxicities related to their treatment. Patients developed other acute side effects with radiation therapy, like mucositis, ulcers, dysphagia, and diarrhea.

In the current project, there was an increase in grades of dermatitis among patients with treatments, as results depicted that at the fourth week of treatment, 98 (92.5%) patients had mild dermatitis (grades 1 and 2). At the seventh week of treatment, severe dermatitis (grade 4) was observed in 58 (7%) patients. However, a decline in grades of dermatitis post-treatment among all patients was observed, as shown in Table 6. Previous studies held on head and neck cancer treatment with chemo-radiotherapy fully supported our results and findings that acute side effects develop during treatment but improve post-radiation if followed regularly and properly [13].

In terms of xerostomia, after the fourth week of treatment, grade 3 was observed in 10 (9.4%) patients, whereas mild xerostomia (grades 1 and 2) was observed in 86 (81%) patients, respectively. At the seventh week of treatment, severe xerostomia of grade 4 was observed in 57 (53.8%) patients. Post-treatment, a decline in grades of xerostomia among patients was observed, as given in Table 8. Our findings were similar to the results of previous studies that showed a decline in acute side effects after one month of radiation therapy [14,15].

Cisplatin-based regimens are very often used for the radiotherapy of squamous head and neck cancer patients, particularly regimens including cisplatin alone. Almost 17.9% of patients (stage 3) received chemoradiation with cisplatin at a dose of 40 mg/m2 once weekly for seven weeks throughout the treatment period. No mortality was noted in the current study, and good local and regional control was seen. Similarly, in the past, a study compared two different doses of cisplatin (the lower dose of 20 mg/m2/day and the higher dose of 25 mg/m2/day) five days per week for four weeks. They concluded that a lower dose was well tolerated and had a better response among cancer patients. Thus, their findings supported our results that cisplatin at a lower dose is much more effective for a longer period of treatment than at a higher dose [16].

Tumor response was assessed four weeks after treatment completion by means of CT scans. Cisplatin was administered concurrently on a weekly basis as a one-hour intravenous infusion of 40 mg/m2. All patients received vigorous hydration and anti-emetic therapy before cisplatin. Our work was in line with previous research on head and neck cancer patients receiving RT [11].

Abundant studies had reported acute side effects of RT in head and neck cancer patients, but few studies had scrutinized whether they affected cancer treatment responses. In the current project, we assessed acute side effects and clinical signs and symptoms during and immediately after follow-up after four weeks of complete treatment. It was a rare study in the sense that it evaluated acute side effects related to chemo-radiotherapy and treatment response clinically in head and neck cancer patients. This study helped to evaluate the reasons for the increasing burden of head and neck cancer cases in the Pakistani population despite the treatment given. In the present study, patients showed clinical improvement in terms of signs and symptoms, and no deaths were reported both during and after treatment.

Limitations

A small sample size with a limited time frame, financial constraints, and limited human resources added to our limitations. However, more studies with a large sample size and long-term follow-up are recommended to see chronic side effects as well. This study helped us design a protocol for proper management of these side effects in order to reduce the illness, as it added useful insight into the disease.

## Conclusions

It was concluded that 3D-radiation therapy was associated with the development of acute side effects like dermatitis and xerostomia during and immediately after follow-up, although the treatment response was good. Moreover, 40 mg/m2 cisplatin once weekly for seven weeks resulted in better loco-regional control and treatment response among cancer patients with no delay in treatment due to its harmful side effects. Results supported our conclusion, as no mortality was reported during treatment or in the follow-up period, and grades of toxicity improved during the follow-up period. However, patients improved clinically in their signs and symptoms. Thus, a low dose (40 mg/m2) of cisplatin once weekly rather than a higher dose (100 mg/m2) should be given to cancer patients requiring cisplatin in our clinical settings in order to avoid poor compliance due to side effects.
